# Chemical composition and *in vitro* rumen fermentation characteristics of various tropical seaweeds

**DOI:** 10.5455/javar.2023.j731

**Published:** 2023-12-31

**Authors:** Nur Hidayah, Cuk Tri Noviandi, Andriyani Astuti, Kustantinah Kustantinah

**Affiliations:** 1 Graduate School of Animal Science, Universitas Gadjah Mada, Yogyakarta, Indonesia; 2 Department of Animal Science, Faculty of Agriculture, Universitas Tidar, Magelang, Indonesia; 3 Department of Animal Nutrition and Feed Science, Faculty of Animal Science, Universitas Gadjah Mada, Yogyakarta, Indonesia

**Keywords:** Chemical composition, gas production, methane emission, tropical macroalga

## Abstract

**Objective:**

This research aimed to evaluate potential tropical seaweed from Indonesia as an ingredient or supplement feed for ruminants based on chemical composition and *in vitro* rumen fermentation parameters.

**Materials and Methods:**

The seven natural tropical seaweeds (three green and four red species) were collected from Ndrini and Sepanjang Beach, Gunungkidul, Yogyakarta, Indonesia. The experimental design on secondary metabolite profiles used a completely randomized design, and the *in vitro* gas production test used a randomized complete block design with seven seaweed species variances and four replications (blocks) based on rumen fluid collection time. The data obtained was analyzed using analysis of variance (ANOVA), and Duncan‘s Multiple Range Test was used to test the variation in the analysis.

**Results:**

The seven tropical seaweed species have potential as mineral sources for ruminants, except for macromineral (P and S) and micromineral (Cu). The red tropical seaweed has potential as a protein source (*Gelidium spinosum* (S.G.Gmelin) P.C. Silva*, Hypnea pannosa,* and* Acanthopora muscoides* (L.) Bory), and the green seaweed (*Chaetomorpha linum* (O.F. Mull.) Kutz and* Cladopora* sp.) has potential as a crude fiber (CF) source for ruminants. As indicated by secondary metabolites and gas production* in vitro*, the green species (*C. linum* (O.F. Mull.) Kutz and *Enteromorpha compressa*) and red species (*A. muscoides* (L.) Bory and *Gelidium amansii* (J.V. Lamouroux) J.V. Lamouroux) could be degraded in the rumen and had quite high phenolic compounds.

**Conclusion:**

The seven tropical seaweed species have the potential to be an ingredient or supplement feed for ruminants, and there were four species that have the potential to reduce methane emissions.

## Introduction

The Intergovernmental Panel on Climate Change (IPCC) 2022 reported that methane (CH_4_) is a strong and fast trigger of climate variation [1]. Methane is a GHG that has been emitted for over 100 years and has an 80 times more intense effect than carbon dioxide (CO_2_) over 10–20 years from the time it is released into the atmosphere. [2]. The Paris Agreement on Climate Change agreed that preventing the rise in atmospheric temperature does not exceed 2°C, so an effort to decrease CH_4_ emissions is important [3]. Ruminants such as sheep, cattle, and goats donate 17% of total anthropogenic emissions to methane production, with enteric CH_4_ produced by rumen fermentation of feed [4]. CO_2_ and hydrogen (H_2_) gas were produced by enteric fermentation, which plays an essential role in the creation of CH_4_ in the cutback process of archaea microbes throughout methanogenesis [5]. Efforts to reduce methane emissions from ruminants are important considering the emission of methane has two bad effects: it contributes to the production of greenhouse gases and reduces ruminant productivity. Energy from feed 3%–12% is missed as methane [6].

Min et al. [7] reported that several strategies to reduce CH_4_ production in ruminants that have been implemented include the addition of ionophores, chemicals, legumes, essential oils, fats, probiotics, and secondary metabolites (tannins, saponins, halogens, and phlorotannins) to ruminant feed. Recent research reported that seaweed very efficiently reduces CH_4_ emissions in ruminants [7–10] by reason of the content of metabolites, especially the halogen compound. Halogenated compounds, including bromoform, are anti-methane compounds that are able to inhibit the methyl-coenzyme reductase (MCR) enzyme during methanogenesis [11]. The decrease in CH_4_ production will lead to more energy formation via increased volatile fatty acid (VFA) production [12]. Nowadays, the species of seaweed with Asparagopsis genus are the most forceful additive in decreasing enteric CH_4_ production (40%–98%) with low levels (0.2%–2% organic matter (OM)) [9,13–17] tried for another species of seaweed as an alternative CH_4_ mitigation, and the result showed that the addition of 6% and 10% OM of *Bonnemaisonia hamifera* on perennial ryegrass as basal feed reduced 95.4% and 98.8% CH_4_ production with minimal effect on* in vitro* fermentation characteristics.

Studies on the effectiveness of seaweed species in reducing enteric CH_4_ are still limited to subtropical seaweed species, so they need to be expanded to tropical seaweed species. Indonesia is one of the greatest tropical seaweed producers in the world (38.7%) after China (47.9%) [18]. Erniati et al. [19] reported that the Indonesian variance of seaweed species is the greatest in comparison to other nations. The Van Bosse expedition in the Siboga Sea area in 1,899–1,900 revealed about 555 types of seaweed genetic material from about 8,000 types of seaweed in the world, which can be grown well in Indonesia and have excellent quality [20]. The Indonesian Central Statistics Agency [21] reports that Indonesia produced seaweed in 2021, reaching 5,011 million wet tons and becoming one of the world‘s main assemblers of watery seaweed [20]. So far, in Indonesia, seaweed has been used as fresh concrete for the food, medicine, and cosmetic industries [20,22] and is not generally used as animal feed. Besides that, the use and commercialization of seaweed still need to be improved in *Euchema* spp. and *Gracilaria* spp. Therefore, many seaweed species have yet to be explored [23] and have the potential for diversity and availability. Therefore, it is very necessary and useful to evaluate the conceivable use of tropical seaweed from Indonesia with different species as a substitute feed ingredient or feed supplement for ruminants that have the potential to depress enteric CH_4_ emissions.

## Materials and Methods

### Animals

Two Balinese bulls and cows with rumen fistulas (body weights of 350 and 290 kg; 5 years old) from the Faculty of Animal Sciences, Gadjah Mada University, Indonesia, were used as rumen sources for *in vitro* incubation. All experimental procedures were allowed by the Animal Care Board of the Veterinary Medicine Faculty, Gadjah Mada University, Yogyakarta, Indonesia (allowed number: 052/EC-FKH/Eks./2022). The animals are fed a diet that includes elephant grass (*Pennisetum purpureum*) and pollard with a ratio of 60%:40% DM offered twice at 8 a.m. and 5 p.m., and clean water is always available.

### Collection and preparation of seaweed

The natural seaweeds were assembled by picking them out in June 2022 from Ndiri and Sepanjang Beach, Gunungkidul, Yogyakarta, Indonesia. The picked seaweeds were three green species (*Chaetomorpha linum* (O.F. Mull.) Kutz*, Enteromorpha compressa,* and* Cladopora* sp.) and four red species (*G. spinosum* (S.G.Gmelin) P.C. Silva*, Hypnea pannosa, A. muscoides* (L.) Bory, and* Gelidium amansii* (J.V. Lamouroux))*.* Tropical seaweed identification using morphological methods was carried out in the Laboratory of Plant Systematics, Faculty of Biology, Universitas Gadjah Mada, Yogyakarta, Indonesia. The seaweed samples were cleaned with water from sand and other dirt and then dried with a freeze dryer (Buchi, Lyovapor, L-200). After that, we used a hammer mill to grind the freeze-dried samples until a fine powder (80–100 mesh) formed and stored them in a freezer for further analysis in sealed polybags.

### Chemical composition analysis

The chemical profile analysis used powder samples of seven tropical seaweed species, including proximate analysis, Van Soest analysis, macro and microminerals, and secondary metabolite contents. The proximate analysis consisted of dry matter (DM), ash, organic matter (OM), crude protein (CP), ether extract (EE), crude fiber (CF), and nitrogen-free extract (NFE) measured according to AOAC [24]. The DM concentration was calculated after the sample was dried at 105°C, and ash was measured as residual after burning at 550°C. The amount of OM was calculated as [100−ash], and CP was computed as *N* (Kjeldahl method analysis) × 6.25. The EE was calculated after extracting the sample using the Soxhlet method and drying at 105^o^C. Meanwhile, the CF was evaluated with the boiled sample using a solution of H_2_SO_4_ (sulphuric acid) and continued to be boiled with a solution of sodium hydroxide (NaOH) for 30 min at 300^o^C, then dried at 105^o^C. The amount of NFE was estimated as [100−(ash+CP+EE+CF)]. Van Soest’s analysis, measured according to [25], consisted of neutral detergent fiber (NDF). The sample was evaluated by boiling it in an NDF solution for 15 min at 300^o^C and then boiling it in an ADF solution for 15 min at 300^o^C to evaluate acid detergent fiber (ADF). Hemicellulose was calculated as [NDF-ADF]. The bomb calorimeter (Parr, 6400 Calorimeter, Parr Instrument Company, Moline, IL, USA) used for gross energy analysis and mineral profile were evaluated using an AAS spectrophotometer (AA-610S, Shimadzu Company, Kyoto, Japan) that was analyzed at the Assessment Institute for Agricultural Technology (BPTP) in Yogyakarta, Indonesia. All tests were performed in duplicate for each seaweed species.

The secondary metabolite analysis included phenol, tannin, phlorotannin, flavonoid, and bromoform. Preparing the extracts according to the method described by Abdulrazak and Fujihara [26], who described methanol as the solvent. Briefly, 200 mg of dried seaweed mill was expanded in 10 ml of methanol solvent and produced in a platform incubator shaker series (Innova 42, New Brunswick, Eppendorf AG, DE) for 90 min at 130 rpm and at 30°C for the extraction process. The mixture sample was spun for 20 min at 4°C at 3000 g and transferred to another tube as much as possible without disturbing the residue for phenol, tannin, and flavonoid analysis. The Folin–Ciocalteau measured phenol and tannin according to Makkar [27]. Tannic acid (Sigma–Aldrich) solutions in the 20–100 µg/ml range were used for the standard curve (*y* = 0.0108*x* + 0.0102, *r*^2^ = 0.99), and the absorbent was read at a wavelength of 725 nm. Measuring the flavonoid content using the Dowd method, as adapted by Arvouet–Grand and Vennat [28], Quercetin (Sigma–Aldrich) solutions in the 20–100 µg/ml range were used for the standard curve (*y* = 0.002934*x*–0.032, *r*^2^ = 0.99), and the absorbent was read at a wavelength of 415 nm. Bromoform content was measured according to the Romanazzi et al. [29] method. Up to 100 mg of lyophilized seaweed powder was transferred into a 15-ml screw-capped polypropylene spin tube and extracted twice with a methanol solution of as much as 10 ml in an ultrasonic water bath at 5°C–6°C for 30 min. The extract was spun (3,000 g for 10 min) before collecting the supernatant. Dilute up to 0.1 mL of the composite to 10 mL with methanol to bring the extract within the calibration range. Samples of these diluted extracts were analyzed by GC-MS using an Agilent 8890 gas chromatograph equipped with a single quadrupole mass analyzer (Agilent 5977B) and an Agilent 19091N-2361 HP-INNOWAX silica capillary column (60 m × 0.25 mm i.d., 0.50 μm film thickness). Bromoform was determined by its characteristic fragment ion (m/z: 170.8, 172.8, 174.8, 251.8) and quantified by comparison with a bromoform standard curve of bromoform (0.025–2.50 µg/ml, *y* = 223.59*x*+14.96, *r*^2^ = 0.99) using certified reference material (Sigma–Aldrich).

### In vitro evaluation, sampling, and calculation

We used the Menke and Steingass [30] method for *in vitro* evaluation. All seaweed samples (powder form) were used as a substrate allocated in 100 ml glass syringes (Haberle Labortechnik, Lonsee, Germany) with as much as 200 mg DM and added to a buffer solution and 30 ml rumen liquid (2:1 ratio vol/vol) at 39°C for 72 h incubation in anaerobic conditions. Rumen liquid was composed of the two Balinese cows with rumen fistulas before morning feeding, pooled, and transported immediately to the laboratory in thermos flasks. The rumen liquid was filtered over four layers of gauze and composited with a solution of buffer. A total of 36 glass syringes for *in vitro* incubation runs were conducted: 28 glass syringes for the samples, four glass syringes for blanks (without substrate) to appropriate the gas production for gas that releases from endogenic yield, and four glass syringes for standard (the seaweed substrate replaced with Pangola grass) as an indicator *in vitro* incubation process. The gas production was evaluated at 2, 4, 8, 12, 24, 48, and 72 h. Samples for methane gas production analysis (10 ml) were taken from the aliquot after 24 h incubation and stored in a vacuum tube (Kang Jian, China). Gas samples were measured for methane emissions using the Fievez et al. [31] method. In comparison, samples for fermentation characteristics (pH, VFA, NH_3_, and rumen microbial protein) were analyzed after 72 h incubation, then centrifuged for 15 min at 3,000 rpm and stored in the freezer for further analysis in a 1.5 ml microtube.

Calculation of the gas production using the Neway program [32] based on the equalization *Y* = a+b(1-e^-ct^). The pH was determined with a pH meter (Hanna pH-meter portable, Hanna Instruments, USA). Gas samples were measured for methane emissions using gas chromatography (GC 14B, Shimadzu Croporation, Kyoto, Japan, with a Paropak column (50 m × 0.2 mm × 0.3 μm) and FID detector). A total of 1 ml of supernatant was added to 20% (200 µl) of meta-phosphoric acid in a tube, centrifuged at 3,000 rpm for 10 min, and then the resilient was taken and analyzed using gas chromatography (GC 2010 Plus, Shimadzu Croporation, Kyoto, Japan, HP-FFAP column (50 m × 0.2 mm × 0.3 μm) and FID detector) for VFA concentration measurement. Analysis of ammonia used 1 ml of supernatant and measured based on the indophenol reaction (reaction between ammonia and sodium phenate) as explained by Chaney and Marbach [33] with a spectrophotometer at wavelengths of 750 nm and a standard ((NH_4_)_2_ SO_4_, (Merck) curve (*y* = 0.028018*x*-0.01108, *r*^2^ = 0.99). For microbial protein measurements, 1 ml of supernatant was spun at 10,000 rpm for 10 min, and the residue and added microbial protein solution were analyzed with a spectrophotometer at wavelengths of 630 nm and the standard (BSA/Bovine Serum Albumin, Sigma) curve (*y* = 2.5075*x* + 0.0824, *r*^2^ = 0.99), according to the [34] method.

### Research design and statistical analysis

Chemical composition (ex: secondary metabolites) data were analyzed definitively by considering the mean value of the collected data. The analysis of the secondary metabolite design used a completely randomized design with seven seaweed species (*C. linum* (O.F. Mull.) Kutz*, E. compressa,* and* Cladopora* sp., *G. spinosum* (S.G.Gmelin) P.C. Silva*, H. pannosa, A. muscoides* (L.) Bory, and* G. amansii* (J.V.Lamouroux) and four replications in each treatment. Meanwhile, the experiment *in vitro* rumen fermentation design used a randomized complete block (time of rumen fluid collection) design with seven seaweed species and four replications in each treatment. The data were calculated statistically using analysis of variance (ANOVA), and Duncan‘s multiple range test examined the differences among the means. Statistical analysis was performed using IBM SPSS Statistics version 26.

## Results

### Nutrient profile

*Cladopora* sp. had the lowest OM (36.75% DM) and highest ash (63.25% DM). All the seaweed species in this research had more than 30.00% DM of ash. Red seaweed generally had greater CP (14.53%–22.48% DM) than green seaweed (9.73%–15.44% DM). The EE of all seaweed was less than 1.00% DM; the highest EE in* C. linum* (O.F.Mull.) Kutz and *H. pannosa* were at 0.77% and 0.72% DM, respectively, while the lowest EE in *E. compressa* and *G. spinosum* (S.G.Gmelin) P.C. Silva was at 0.16% and 0.10% DM, respectively. The green seaweed had greater CF (6.23%–22.84% DM) than the red seaweed (6.48%–10.64% DM) and the highest CF in *C. linum* (O.F.Mull.) Kutz at 22.84% DM. *Enteromorpha compressa* had the lowest CF (6.23% DM), highest NFE (43.98% DM), and hemicellulose (44.62% DM) in green seaweed. In contrast, red seaweed has the highest NFE in *A. muscoides* (L.) Bory at 35.57% DM. *Cladopora* sp. had the highest NDF (75.26% DM), ADF (47.26% DM), and gross energy (2,958 Cal/gm DM) in green seaweed. *Hypnea pannosa* had the highest NDF (75.26% DM), ADF (47.26% DM), and gross energy (2,486 Cal/gm DM) in red seaweed (Table 1).

### Mineral profile

In green seaweed, *E. compressa* had the highest macromineral (P, Na, Mg, and S at 0.20%, 11.50%, 2.41%, and 0.21% DM, respectively) and micromineral (Fe, Mn, and Zn at 4,503.67, 201.38, and 566.67 mg/kg DM, respectively) and the lowest heavy metal Pb at 6.57 mg/kg DM. *Cladopora* sp. had the highest Na at 2.79% DM and Cu at 11.46 mg/kg DM. *Chaetomorpha linum* (O.F.Mull.) Kutz had the highest K at 2.36% DM. Meanwhile, in red seaweed, *H. pannosa* had the highest macromineral (Na, Ca, and Mg at 3.75%, 12.21%, and 0.83% DM, respectively) and micromineral Mn at 143.79 mg/kg DM. *Acanthopora muscoides* (L.) Bory had the highest P and S at 0.11% and 0.17% DM, respectively, Zn at 1,700.00 mg/kg DM, and no detection of heavy metal Pb. *Gelidium amansii* (J.V.Lamouroux) had the highest P and K at 0.12% and 7.78% DM, respectively, Cu at 31.38 mg/kg DM, and no detection of heavy metal Pb too.* Gelidium spinosum* (S.G.Gmelin) P.C.Silva had the highest Fe at 2,066.94 mg/kg DM (Table 2). This study‘s seaweed is a poor source of P and S with low heavy metals Pb and Cd.

**Table 1.** Nutrient composition (%DM, excepting DM content) and gross energy (Cal/gm DM) of seven tropical seaweed species.

Chemical composition	Green seaweed			Red seaweed			
	GS1	GS2	GS3	RS1	RS2	RS2	RS3
DM	7.29	7.61	14.17	10.68	8.44	6.12	10.12
OM	60.90	65.81	36.75	60.46	50.71	65.66	51.75
Ash	39.10	34.19	63.25	39.54	49.29	34.34	48.25
CP	11.19	15.44	9.73	20.15	19.91	22.48	14.53
EE	0.77	0.16	0.46	0.10	0.72	0.28	0.20
CF	22.84	6.23	16.50	10.64	6.48	7.33	7.11
NFE	26.10	43.98	10.06	29.57	23.61	35.57	29.91
NDF	46.92	69.55	75.26	58.64	62.81	57.58	52.96
ADF	15.63	24.93	47.26	14.49	25.22	16.28	4.42
Hemicellulose	31.30	44.62	28.00	44.15	37.59	41.30	48.55
Gross energy	2,357	2,104	2,958	2,239	2,486	2,671	2,276

DM: dry matter, OM: organic matter, CP: crude protein, EE: ether extract, CF: crude fiber, NFE: nitrogen-free extract as 100-(ash+CP+EE+CF), NDF: neutral detergent fiber, ADF: acid detergent fiber, GS1, *C. linum* (O.F.Mull.) Kutz; GS2, *E. compressa*; GS3, *Cladopora* sp.; RS1, *G. spinosum* (S.G.Gmelin) P.C.Silva; RS2, *H. pannosa*; RS3, *A. muscoides* (L.) Bory; RS4, *G. amansii* (J.V.Lamouroux) J.V.Lamouroux

### Secondary metabolite profile

The different species of tropical seaweed had different percentages of secondary metabolite content (*p* < 0.01), as presented in Table 3. All the seaweed samples in this experiment had a richer flavonoid content than phenol and tannin contents. The highest flavonoid content (*p* < 0.01) was found at *C. linum* (O.F.Mull.) Kutz (9.02 mg quecetin per gm of DM). The green seaweed had higher flavonoids than the red seaweed. The lowest (*p* < 0.01) secondary metabolite content in red seaweed at *H. pannosa* (phenol and tannin: 0.53 and 0.51 mg tannic acid per gm of DM, and flavonoid: 1.54 mg quecetin per gm of DM) and in green seaweed at *Cladopora* sp. (phenol and tannin: 0.56 and 0.54 mg tannic acid per gm of DM) except the flavonoid, the lowest at *E. compressa* (3.39 mg quecetin per gm of DM). The bromoform was not detected in all seaweeds in the current work, possibly due to the percentage of bromoform content being too low, losses due to evaporation, or these species containing other brominated halomethane compounds (e.g., dibromoacetic acid, dibromochloromethane, and bromochloroacetic acid). De Bhowmick and Hayes [35] stated that bromoform is a volatile compound that easily evaporates.

**Table 2.** Mineral content of seven tropical seaweed species.

Mineral	Green seaweed			Red seaweed			
	GS1	GS2	GS3	RS1	RS2	RS2	RS3
Macromineral (% DM)							
P	0.03	0.20	0.06	0.09	0.07	0.11	0.12
K	2.36	0.90	2.07	4.26	1.06	3.82	7.78
Na	1.55	1.02	2.79	0.71	3.75	0.66	1.36
Ca	0.41	11.50	10.46	1.54	12.21	1.80	1.74
Mg	0.43	2.41	0.68	0.40	0.83	0.61	0.34
S	0.05	0.21	0.10	0.12	0.07	0.17	0.14
Micromineral (mg/kg DM)							
Fe	1,643.39	4,503.67	2,570.86	2,066.94	1509.80	1,228.71	486.85
Mn	39.03	201.38	188.62	55.41	143.79	133.09	112.52
Cu	6.16	6.57	11.46	7.76	4.36	5.32	31.38
Zn	500.00	566.67	309.09	214.29	625.00	1,700.00	37.93
Heavy metals (mg/kg DM)							
Pb	15.41	6.57	11.46	14.41	20.70	nd	nd
Cd	1.03	3.28	3.13	2.22	3.27	2.13	2.16

Mineral (P: phosphor, K: potassium, Na: sodium, Ca: calcium, Mg: magnesium, S: sulfur, Fe: ferrum, Mn: manganese, Cu: cuprum, Zn: zinc, Pb: lead, Cd: cadmium); GS1, *C. linum* (O.F.Mull.) Kutz; GS2, *E. compressa*; GS3, *Cladopora* sp.; RS1, *G. spinosum* (S.G.Gmelin) P.C.Silva; RS2, *H. pannosa*; RS3, *A. muscoides* (L.) Bory; RS4, *G. amansii* (J.V.Lamouroux) J.V.Lamouroux

**Table 3.** Secondary metabolites content of seven tropical seaweed species (mg/gm DM).

Variables	Green Seaweed			Red Seaweed			
	GS1	GS2	GS3	RS1	RS2	RS2	RS3
Phenol	0.90^bc^ ± 0.14	0.82^b^ ± 0.08	0.56^a^ ± 0.06	0.92^bc^ ± 0.06	0.53^a^ ± 0.09	0.98^c^ ± 0.12	0.96^bc^ ± 0.12
Tannin	0.88^b^ ± 0.14	0.80^b^ ± 0.08	0.54^a^ ± 0.06	0.87^b^ ± 0.06	0.51^a^ ± 0.09	0.94^b^ ± 0.12	0.93^b^ ± 0.11
Flavonoid	9.02^d^ ± 0.47	3.39^c^ ± 0.40	3.70^c^ ± 0.38	1.88^a^ ± 0.11	1.54^a^ ± 0.17	2.93^b^ ± 0.16	2.80^b^ ± 0.20
Bromoform	nd	nd	nd	nd	nd	nd	nd

Note: Means in the same lines with different superscripts differ high significantly (*p* < 0.01); GS1, *C. linum* (O.F.Mull.) Kutz; GS2, *E. compressa*; GS3, *Cladopora* sp.; RS1, *G. spinosum* (S.G.Gmelin) P.C.Silva; RS2, *H. pannosa*; RS3, *A. muscoides* (L.) Bory; RS4, *G. amansii* (J.V.Lamouroux) J.V.Lamouroux, nd: not detected

### Gas production and methane emissions

The gas production was significantly different (*p* < 0.01) among the treatments at all observed incubation times except at 12 h. *Enteromorpha compressa* has the highest gas production of all seaweeds after 72 h of incubation. *Hypnea. pannosa* has the highest gas production at 2–4 h of incubation. *Cladopora* sp. has the lowest gas production at all observed incubation times of all seaweeds. The result of gas production was linear, and the gas production curve indicated that the most easily degraded fraction (a) was on *H. pannosa*, a potentially degraded fraction (b) was on *E. compressa*, and the rate of gas production of the b fraction (c) was on *Cladopora* sp. Meanwhile, the highest total fraction degraded (a+b) values were on *E. compressa* for green seaweed and *H. pannosa* for red seaweed*.* The methane gas production was highest after 24 h of incubation at *H. pannosa* (Tables 4 and 5)*.*

### Rumen fermentation characteristics

Rumen fermentation characteristics of different tropical seaweed species were significantly different (*p* < 0.05) except for pH value and rumen microbial protein. The pH value in this study was 6.99–7.13, which was still considered normal. Dehority [36] stated that the normal rumen pH was 5.40–7.80. Meanwhile, the rumen microbial protein was 5.88–9.53 mg/100 ml. The red seaweed, *A. muscoides* (L.) Bory and *G. amansii* (J.V.Lamouroux), had high total VFA, propionate, and low acetate, butyrate, and A/P ratio (115.06 mM, 18.00%, 71.75%, 10.25%, 3.99, and 120.15 mM, 16.78%, 73.18%, 10.05%, 4.37, respectively). For red seaweed, the low total VFA, propionate, high acetate, butyrate, and A/P ratio were found at *H. pannosa* (59.45 mM, 13.74%, 73.44%, 12.81%, and 5.41), and for green seaweed, they were found at *Cladopora* sp. (59.31 mM, 14.86%, 74.38%, 10.76%, and 5.04). *Acanthopora muscoides* (L.) Bory had the highest NH_3_ concentration (31.86 mg/100 ml) and the lowest at *Cladopora* sp. (22.22 mg/100 ml). The data presented in Table 6

**Table 4.** Gas production (ml/200 mg DM) of seven tropical seaweed species over 72 h.

Incubation (Hours)	Green Seaweed			Red Seaweed			
	GS1	GS2	GS3	RS1	RS2	RS2	RS3
2	2.01^a^ ± 0.47	1.41^a^ ± 0.31	1.72^a^ ± 0.31	3.42^b^ ± 0.51	5.52^c^ ± 1.91	2.18^a^ ± 0.30	2.11^a^ ± 0.52
4	2.68^ a^ ± 0.72	3.01^a^ ± 0.33	2.57^a^ ± 0.03	5.20^b^ ± 0.68	7.10^c^ ± 1.93	3.86^ab^ ± 0.59	4.04^ab^ ± 0.63
8	4.29^a^ ± 0.90	7.34^b^ ± 1.67	4.28^a^ ± 0.57	7.94^b^ ± 0.23	7.28^b^ ± 3.06	6.91^ab^ ± 1.68	6.14^ab^ ± 1.14
12	6.32 ± 0.76	10.13 ± 1.61	6.82 ± 2.16	10.12 ± 0.51	9.53 ± 3.19	8.70 ± 2.00	7.89 ± 0.90
24	11.75^ab^ ± 1.26	16.92^d^ ± 1.89	10.81^a^ ± 2.43	14.78^bcd^ ± 1.52	15.98^cd^ ± 2.43	12.90^abc^ ± 2.72	12.09^ab^ ± 2.16
48	17.68^ab^ ± 2.78	26.31^c^ ± 1.75	13.77^a^ ± 3.02	19.43^ab^ ± 2.25	22.48^bc^ ± 0.86	17.75^ab^ ± 3.13	18.59^ab^ ± 5.42
72	21.21^ab^ ± 4.15	31.82^c^ ± 2.08	15.95^a^ ± 3.74	22.17^b^ ± 2.88	25.29^b^ ± 1.16	19.41^ab^ ± 4.58	21.20^ab^ ± 6.62

^a,b,c,d^Means in the same lines with different superscripts differ significantly (*p* < 0.05); GS1, *C. linum* (O.F.Mull.) Kutz; GS2, *E. compressa*; GS3, *Cladopora* sp.; RS1, *G. spinosum* (S.G.Gmelin) P.C.Silva; RS2, *H. pannosa*; RS3, *A. muscoides* (L.) Bory; RS4, *G.elidium amansii* (J.V.Lamouroux) J.V.Lamouroux

**Table 5.** Gas production over 72 h incubation (a, b, c, and a+b fractions) and methane emission of different tropical seaweed species.

Variables	Green Seaweed			Red Seaweed			
	GS1	GS2	GS3	RS1	RS2	RS2	RS3
a (ml/200 mg DM)	0.46^a^ ± 0.60	0.40^a^ ± 1.75	0.54^a^ ± 0.37	2.13^ab^ ± 0.90	3.85^b^ ± 1.44	1.10^a^ ± 0.62	1.54^a^ ± 1.12
b (ml/200 mg DM)	26.04^b^ ± 7.16	36.87^c^ ± 1.31	15.59^a^ ± 3.26	21.12^ab^ ± 4.12	23.91^b^ ± 5.39	20.34^ab^ ± 6.38	21.33^ab^ ± 6.53
c (ml/hours)	0.025^a^ ± 0.00	0.030^ab^ ± 0.00	0.040^c^ ± 0.01	0.043^c^ ± 0.01	0.035^b^ ± 0.01	0.040^c^ ± 0.01	0.035^bc^ ± 0.01
a+b (ml/200 mg DM)	26.07^b^ ± 6.97	37.27^c^ ± 2.14	16.74^a^ ± 3.79	23.25^ab^ ± 3.27	26.60^b^ ± 3.31	22.36^ab^ ± 7.20	22.86^ab^ ± 7.43
CH_4.24_(ml/gm DM)	6.59^a^ ± 2.32	6.45^a^ ± 1.52	7.96^a^ ± 0.94	6.63^a^ ± 1.98	11.39^b^ ± 2.26	6.49^a^ ± 2.56	7.75^a^ ± 3.13

^a,b,c^Means in the same lines with different superscripts differ high significantly (*p* < 0.01)); GS1, *C. linum* (O.F.Mull.) Kutz; GS2, *E. compressa*; GS3, *Cladopora* sp.; RS1, *G. spinosum* (S.G.Gmelin) P.C.Silva; RS2, *H. pannosa*; RS3, *A. muscoides* (L.) Bory; RS4, *G. amansii* (J.V.Lamouroux) J.V.Lamouroux, a: gas production from easily degraded fraction, b: gas production from potentially tainted fraction, c: rate of gas production of b fraction, a+b: total fraction degraded and fermented, CH_4.24_: methane gas production on 24 h incubation

## Discussion

### Nutrients composition

The DM of all of the seaweed in this work (6.12%–14.17%) is lower than the DM of grasses that are usual for ruminant feed (*Pennisetum purpuphoides* at 17.82% and *Pennisetum purpureum* cv. (GU) at 24.10%). The low DM of seaweed was similar to the result reported by Ahmad [37]. The DM of 15 seaweeds from Semporna, Sabah, Malaysia, was around 3.97%–24.05%. The DM of four seaweed species from Tuban, East Java, Indonesia, was around 13.67%–30.59% [38]. Meanwhile, this research‘s seaweed species are rich in mineral content (>30.00% DM). Munoz and Diaz [39] stated that the seaweed mineral content could be 10 times higher than terrestrial plant minerals. This condition is caused by the high concentrations of various minerals in seawater, where seaweed lives. The high mineral content of seaweed reported by Mwalugha [40], seaweed from Kenya‘s ocean waters, was around 13.49%–37.62% dry weight. The red seaweed from Tuban, East Java, Indonesia, contains minerals around 23.42%–65.63% DM [38].

In this study, red seaweed‘s CP is higher than green seaweed‘s. Murata and Nakazoe [41] stated that red seaweed contains the highest CP compared to green and brown seaweed. The CP content varies depending on the seaweed type: brown at 4%–24% DM, green at 9%–33% DM, and red at 8%–47% DM [42]. The different results were also reported by Mwalugha [40], who found that the CP content of red and green seaweed from Kenya was not different (*p* > 0.05) at 11.56% and 10.52% dry weight. Some species of green seaweed, such as *Acrosiphonia* sp. from Bodø, Norway, have a higher CP content (33.30% DM collected in spring and 28.60% DM in autumn). Pirian [43] stated that the CP content in seaweeds is influenced by varying species and seasonal periods. The different CP content could be triggered by the variance in growing environmental variables such as water temperature, nutrient availability, and harvest time [44]. The CP content of red seaweed is almost the same as the CP of coconut meal at 22.86% dry weight [45] and comparable with high-protein plant feeds such as soybean at 39.00% DM [46]. Therefore, the tropical red seaweed in this study has more potential as an alternative ruminant protein source.

**Table 6.** Rumen fermentation characteristic of different tropical seaweed species.

Variables	Green Seaweed			Red Seaweed			
	GS1	GS2	GS3	RS1	RS2	RS2	RS3
pH	7.16 ± 0.26	7.03 ± 0.17	7.18 ± 0.24	7.10 ± 0.15	7.13 ± 0.18	6.99 ± 0.07	6.99 ± 0.03
Total VFA (mM)	63.45^a^ ± 3.85	87.62^c^ ± 2.59	59.31^a^ ± 3.24	73.86^b^ ± 7.94	59.45^a^ ± 4.93	115.06^d^ ± 7.10	120.15^d^ ± 6.03
Acetate (%)	74.38^d^ ± 1.08	73.95^bc^ ± 1.66	72.34^ab^ ± 0.70	72.71^abc^ ± 1.55	73.44^abc^ ± 2.00	71.75^a^ ± 0.14	73.18^abc^ ± 0.47
Propionate (%)	14.86^ab^ ± 1.23	16.00^bc^ ± 1.23	16.14^bc^ ± 0.78	15.75^bc^ ± 1.27	13.74^a^ ± 1.57	18.00^d^ ± 0.62	16.78^cd^ ± 0.80
Butyrate (%)	10.76^ ab^ ± 1.16	10.05^a^ ± 0.62	11.53^b^ ± 0.41	11.55^b^ ± 0.75	12.81^c^ ± 0.60	10.25^a^ ± 0.74	10.05^a^ ± 1.11
Acetate/Propionate	5.04^bc^ ± 0.44	4.65^ab^ ± 0.49	4.49^ab^ ± 0.27	4.65^ab^ ± 0.44	5.41^c^ ± 0.73	3.99^a^ ± 0.13	4.37^ab^ ± 0.19
NH_3_ (mg/100 ml)	25.15^b^ ± 0.4	27.71^cd^ ± 0.6	22.22^a^ ± 1.2	29.12^d^ ± 1.46	28.05^cd^ ± 0.64	31.86^e^ ± 1.25	27.02^c^ ± 0.2
Rumen microbial protein (mg/100 ml)	7.85^ab^ ± 1.42	8.07^ab^ ± 1.49	5.88^a^ ± 0.65	8.75^b^ ± 2.62	8.71^b^ ± 1.38	9.53^b^ ± 1.82	7.69^ ab^ ± 0.34

^a,b,c,d^Means in the same lines with different superscripts differ significantly (*p* < 0.05); GS1, *C. linum* (O.F.Mull.) Kutz; GS2, *E. compressa*; GS3, *Cladopora* sp.; RS1, *G. spinosum* (S.G.Gmelin) P.C.Silva; RS2, *H. pannosa*; RS3, *A. muscoides* (L.) Bory; RS4, *G. amansii* (J.V.Lamouroux) J.V.Lamouroux

The EE content of all seaweed species in this study is less than 1.00% DM (0.10%–0.77% DM). The EE content of seaweed is lower than 3.00% [47]; Molina–Alcaide [48]). In general, the EE content of seaweed is very low at 1.00%–3.00% dry weight because seaweed stores food in the form of carbohydrates, especially polysaccharides. Unlike CP, CF red seaweed is lower than green seaweed. This result is different from what was discovered by Mwalugha [40], that the CF content in red and green seaweed from Mkomani–Kibuyuni–Mtwapa, Kenya, does not have significant differences (*p* > 0.05) at 14.28% and 13.30% dry weight. The different environmental conditions for seaweed growth might be due to influences on the CF content. Siddique [49] explained that natural situations (nutrient uptake, salinity, and water transparency for the synthesis of NFE) influence CF levels in seaweed. The seaweed’s CF content in this research, except for *C. linum* (O.F. Mull.) Kutz and *Cladopora* sp., is almost similar to cereal grains (barley, corn, oat, and triticale at 2.90%–11.94% DM), as described by Marin [50]. The highest NFE in *E. compressa* was the same, as discovered by Mwalugha [40] that green seaweed from Kenya (*Ulva lactuca*) was 46.11% dry weight.

The content of NDF and ADF from all tropical seaweed in this experiment was higher than that of subtropical seaweed conveyed by Bikker [44] and Rjiba–Ktita [51]. Therefore, the tropical seaweed in this study can be considered a good source of fiber (NDF and ADF), as stated previously by Lahaye [52]. When tropical seaweeds were compared with terrestrial plants as ruminant fiber sources, the NDF, ADF, and hemicellulose of all seaweed in this study (except *Cladopora* sp.) were lower than those of 226 forages (grasses and legumes) that have NDF at 66.62%–76.39%, ADF at 29.94%–43.41%, and hemicellulose at 28.98%–33.98% DM [53].

### Mineral profile

The seaweeds in this experiment have tolerable macromineral (K, Na, Ca, and Mg) and micromineral (Fe, Mn, and Zn) for ruminant requirements. Feeding ruminants with seaweeds aims to add other mineral sources, especially for its macromineral (P and S) and micromineral Cu, except *Cladopora* sp. and *G. amansii* (J.V.Lamouroux) J.V.Lamouroux. Meanwhile, the seaweed from Tuban East Java Indonesia Ocean water contains insufficient Cu and Zn mineral requirements for ruminants [38]. Excessive seaweed use in ruminant feed requires attention to avoid toxicity to ruminants. The National Research Council [54] advised that the optimum tolerable dietary levels of K, Ca, Mg, Fe, Mn, and Zn for sheep and cattle were at 2.00%, 1.50%, and 0.60% and 500.00 and 2000.00 mg/kg DM, respectively.

Seaweed‘s heavy metals (Pb and Cd) concentration in Gunungkidul, Yogyakarta, Indonesia, is lower than that in Tuban, East Java, Indonesia. This condition indicates that the ocean in Gunungkidul, Yogyakarta, Indonesia, is cleaner and has fewer heavy metal pollutants than the ocean in Tuban, East Java, Indonesia. Deemy [55] stated that FDA CVM CY15-17 regulation controlled the scope of heavy metal levels in livestock feed for Pb at 0.0–12.2 mg/kg and Cd at 0.0–1.40 mg/kg. The use of seaweed in this research (*C. linum* (O.F.Mull.) Kutz, *G. spinosum* (S.G.Gmelin), P.C.Silva, and *H. pannosa*) for ruminant feed needs to pay attention to the Pb heavy metals. Only *C. linum* (O.F.Mull.) Kutz has a lower Cd concentration than the Cd standard level from FDA CVM CY15-17 regulation.

Meanwhile, the maximum tolerance of Cd suggested by the National Research Council [58] was 25 mg/kg in the diet for a few days. The research meta-analysis by Ribeiro et al. [56] described excess metal Cd and Pb as declining sperm quality with high production of oxidative metabolites. The high metals Cd and Pb could damage biological molecules due to excessive ROI production, degeneration of enzymes and receptors by the protein that contains thiol, and mineral effects on the physiology of sperm, potentially damaging male fertility. Guvvala et al. [57] stated that a high level of astuteness or a low level of chronic risk to the contaminants Pb in animals could lower reproductive efficiency.

Some research has confirmed that seaweed can replace mineral sources in ruminant feed. The replacement of limestone with calcareous marine seaweed on the diet at 0.42% and 0.47% DM increased the concentration of phosphor in the serum of postpartum dairy cows [58]. The use of calcareous marine seaweed obtained from *Lithothamnium calcareum* to replace calcium carbonate and calcium propionate for oral Ca supplementation in dairy heifers showed a faster peak of blood total concentration of Ca in comparison to others [59].

### Secondary metabolite profile

The different percentage of tropical seaweed secondary metabolite content in this research was due to the different seaweed species. Dominguez [60] stated that the variance of metabolite composition in seaweed is affected by genetics (species) and environment (e.g., location, nutrient, salinity, and light). The genetics of green seaweed and the environment for seaweed cultivation in this research might be stimulated by the enzymes required for the biosynthesis of flavonoids. As a result, the percentage of flavonoids is higher than the phenol and tannin content, and green seaweed is higher than red seaweed. ArokiaRajan et al. [61] collected seaweed from Rameshwaram, Mandapam region, India. They showed that green seaweed (*Ulva fasciata*) had the highest flavonoid content compared to brown seaweed (*Padina gymnospora*) and red seaweed (*Gracileria edulis*) if extracted with acetone and a mixture of acetone and ethanol (1:1). Besides that, all of the seaweed had the highest flavonoid, total phenol, and tannin content. A similar result was found by Egodavitharana et al. [62]. The green seaweed (*Ulva lactuca* and *Ulva fasciata,* which was poised from Dickwella, Sri Lanka) had higher total flavonoid (9.16 and 8.38 mg rutin equivalent per gm of dry weight, respectively) than total phenolic (0.80 and 0.85 mg gallic acid per gm of dry weight, respectively) content. A different result was identified by La Macchia Pedra et al. [63] who did *in vitro* cultivation for 35 days of the red seaweed (*Kappaphycus alvarezii*) from the Seaweed Area of Marine Shrimp Laboratory (LCM-Federal University of Santa Catarina, Brazil) in sterilized seawater enriched with 50% von Stosch solution at 25°C ± 1°C, under 200 ± 10 μ·mol photons min^−2^ sec^−1^, 12 h photoperiod, 35‰ salinity and continuous aeration showed higher total phenolic content (38 μg galic acid per gm of dry biomass) than flavonoid (7 μg quecetin per gm of dry biomass).

### Gas production and methane emissions

During 72 h of incubation, the gas production in this study was 15.95–31.82 ml/200 mg DM. The same results were discovered in the previous studies, which claimed that the seaweed gas production was 28.50–36.63 ml/200 mg DM [64]. This result determined that seaweed OM degradation in the rumen was relatively low because the gas production was less than 60 ml/200 mg DM. The low gas production in seaweed might be due to the large ash content, reducing the OM. The high NDF and ADF content can also influence the low gas production. Rjiba–Ktita et al. [51] reported a similar result to this study: gas production at 24 h incubation on green macroalgae (*C. linum*) was 76 ml/gm DM (13.20 ml/200 mg DM). The reduced gas production was due to the high ash and low OM content* of C. linum* at 31.90% and 68.10% DM, respectively. In a study on *Ulva sp.* (green seaweed) conducted by Ray and Lahaye [65], soluble fiber fractions in seaweed containing ions (such as calcium) can form gels, which may disturb their degradation in the rumen. Circuncisao et al. [66] stated that minerals would interact with seaweed fiber (several polysaccharides), such as alginate and agar or carrageenan, which form insoluble complexes.

*Enteromorpha compressa* had the highest gas production for green seaweed. It might be due to *E. compressa* having the highest soluble components (CP at 15.44% DM and NFE at 43.98% DM) and hemicellulose (44.62% DM), but the lowest CF (6.23% DM). The rumen microbe did not quickly degrade the NFE *E. compressa* in early incubation, but after 12-h incubation, the NFE and hemicellulose degraded faster, as did CP. This is because protein is a very easily degraded component in the rumen, except for proteins protected using some materials or compounds.

*Hypnea pannosa* has the highest gas production for red seaweed, which might be due to its low CF (6.48% DM) and secondary metabolites (phenols and tannins: 0.53 and 0.51 mg tannic acid per gm DM and flavonoids: 1.54 mg quecetin per gm DM), although it has the highest NDF (75.26% DM) and ADF (47.260% DM) content. Ray and Lahaye [65] and Aquino et al. [67] reported that unlike terrestrial plants (cell walls are mainly made of hemicellulose, cellulose, and lignin), seaweed cell walls contain high amounts of sulfated polysaccharides. Red seaweed contains polysaccharides such as carrageenan, agar, agarose, agaropectin, and porphyran that contain lots of β-D-galactose units [68], which are the soluble fiber fermented in the digestive tract. It is suspected that the cell wall components of *H. pannosa* can be degraded by rumen microbes. The rumen microbiota has a broad enzyme repertoire that can hydrolyze seaweed polysaccharides into simpler sugars and other intermediate products from the fermentation process, which are used for the rumen microbes‘ growth and metabolic activity [69]. In addition, the increased gas production in *H. pannosa* is caused by the low content of secondary metabolite compounds that do not inhibit the degradation of seaweed in the rumen.

Meanwhile, *Cladopora* sp. also had the highest NDF (75.26% DM) and ADF (47.26% DM) but had the lowest gas production. This result may be because *Cladopora* sp. is a green seaweed with cell walls containing ulvan, which has many *β*-(1 → 4) glycosidic bonds found in the cellulose [70]. Cellulose is one of the dietary fibers belonging to insoluble fiber, making it hard to degrade and ferment [71]. This condition caused *Cladopora* sp. to be difficult to hydrolyze and ferment in the rumen. Kulivand and Kafilzadeh [72] reported that gas production has a negative interaction with ADF content (*r* = -0.60; *p* < 0.05). Besides that, the highest mineral content in *Cladopora* sp. (63.25% BK) also affects the low gas production.

*Hypnea pannosa* had the highest CH_4_ production at 24 h of incubation (11.39 ml/gm DM). It may be due to this study‘s lower secondary metabolites of *H. pannosa* (Table 5). They are not high enough to reduce CH_4_ production and the high content of NDF and ADF. Lee–Rangel et al. [73] explained that there is ample evidence showing that seaweed secondary metabolites can reduce rumen CH_4_ production during enteric fermentation. Total phenol (*r* = -0.97) and tannins (*r* = -0.95) had an opposing correlation (*p* < 0.01) with CH_4_ production. Lee–Rangel et al. [73] also explained that CH_4_ production has a positive interaction (*p* < 0.05) with NDF content (r = 0.81). The high NDF will change the proportion of VFA while increasing the portion of acetic acid, which produces hydrogen (H_2_) gas as a substrate in methanogenesis reactions. Uniform results were stated by Maccarana et al. [74], indicating that reducing NDF will increase gas production and reduce CH_4_ emissions.

### Rumen fermentation characteristics

*Acanthopora muscoides* (L.) Bory and *G. amansii* (J.V. Lamouroux) from red seaweed provide higher energy for ruminants. Cheong [69] stated that VFAs are essential components for rumen fermentation and digestion and act as energy sources for ruminant productivity. The high VFA and propionate content of *A. muscoides* (L.) Bory and *G. amansii* (J.V.Lamouroux) might be because they had high soluble carbohydrate content (NFE: 35.57% and 29.91% DM, hemicellulose: 41.30% and 48.55% DM) and low insoluble carbohydrate content (ADF: 16.28% and 4.42% DM). Meale et al. [75] stated that the high soluble carbohydrate content is offered to facilitate propionate creation in the rumen, lower ruminal pH, intruded methanogen growth, and reduce methane production per unit OM fermented. The high nonfiber carbohydrate content in feed proportionally reduced the acetate-to-propionate ratio and spread the propionate content [76]. In contrast, *H. pannosa* for red seaweed and *Cladopora* sp. for green seaweed had high NDF and ADF content (61.81%, 75.26%, and 47.26%, 25.22% DM, respectively).

The highest NH_3_ concentration at *A. muscoides* (L.) Bory (red seaweed) and the lowest at *Cladopora* sp. (green seaweed) were 31.86 and 22.22 mg/100 ml, respectively. This condition might be due to the effect of the CP content in the seaweed. *Acanthopora muscoides* (L.) Bory had the highest CP (22.48% DM), and *Cladopora* sp. had the lowest CP content (9.73% DM). The same result was reported by [45]. *Porphyra* sp. had the highest CP content (34.70% DM) and NH_3_ concentration (46.10 mg/100 ml), and *Pelvetia canaliculata* had the lowest CP content (9.00% DM) and NH_3_ concentration (19.30 mg/100 ml).

From all variables that were evaluated, the seaweed species that had high soluble components (CP, NFE, and hemicellulose) had high gas production, which indicated that they were more degradable in the rumen. Besides that, it stimulated an increase in VFA and rumen microbial protein, even though it is not yet able to reduce methane gas production. The seaweed that had high levels of CF, ADF, and minerals had low gas production, which indicated that it was quite difficult to degrade rumen microbial. This condition stimulated an increase in methane gas production and a decrease in VFA and rumen microbial protein. Methane gas production is influenced by the phenolic compound. As a result of the study, the seaweed species that had the lowest phenolic compounds had the highest methane gas production. However, seaweed, which has the highest phenolic compound, does not produce the lowest methane gas production.

## Conclusion

The seven tropical seaweed species have potential as mineral sources for ruminants except for macromineral (P and S), micromineral (Cu), and less heavy metal (Pb and Cd) concentrations. The red tropical seaweed has the potential as a protein source (*G. spinosum* (S.G.Gmelin) P.C. Silva*, H. pannosa,* and* A. muscoides* (L.) Bory), and the green seaweed (*C. linum* (O.F.Mull.) Kutz and* Cladopora* sp.) has the potential as a CF source for ruminants. As indicated by the secondary metabolites and *in vitro* gas production, the green species (*C. linum* (O.F. Mull.) Kutz and *E. compressa*) and red species (*A. muscoides* (L.) Bory) and *G. amansii* (J.V. Lamouroux) could be degraded in the rumen and had quite high phenolic compounds, which have the potential to reduce methane emission from ruminants. Additional in *vitro* research is needed to evaluate the optimum seaweed admission levels in diets to reduce ruminant methane emissions.

## List of abbreviations

AOAC, Association of Official Agricultural Chemists; AAS, Atomic Absorption Spectroscopy; kg, kilograms; °C, degree Celsius; N, nitrogen; mg, milligram; min, minutes; sec, seconds; rpm, revolutions per minute; g, g-force/gravitation force; µg, microgram; ml, millilitres; µl, microliters; nm, nanometers; h, hours; m, meters; mm, millimetres; μm, micrometers; gm, grams; Cal, calory; mM, milli molars; μmol, micromoles; ROI, Reactive Oxygen Intermediate.
